# The Use of Gut Microbial Modulation Strategies as Interventional Strategies for Ageing

**DOI:** 10.3390/microorganisms10091869

**Published:** 2022-09-19

**Authors:** Ruqaiyyah Siddiqui, Mohammad Ridwane Mungroo, Ahmad M. Alharbi, Hasan Alfahemi, Naveed Ahmed Khan

**Affiliations:** 1 College of Arts and Sciences, American University of Sharjah, Sharjah P.O. Box 26666, United Arab Emirates; 2 Department of Clinical Sciences, College of Medicine, University of Sharjah, Sharjah P.O. Box 27272, United Arab Emirates; 3 Department of Clinical Laboratory Sciences, College of Applied Medical Sciences, Taif University, Taif 21944, Saudi Arabia; 4 Department of Medical Microbiology, Faculty of Medicine, Al-Baha University, Al-Baha 65799, Saudi Arabia

**Keywords:** gut microbiota, microbiota modulation, microbiota development, FMT, faecal microbiota transplantation, prebiotics, probiotics

## Abstract

Gut microbial composition codevelops with the host from birth and is influenced by several factors, including drug use, radiation, psychological stress, dietary changes and physical stress. Importantly, gut microbial dysbiosis has been clearly associated with several diseases, including cancer, rheumatoid arthritis and *Clostridium difficile*-associated diarrhoea, and is known to affect human health and performance. Herein, we discuss that a shift in the gut microbiota with age and reversal of age-related modulation of the gut microbiota could be a major contributor to the incidence of numerous age-related diseases or overall human performance. In addition, it is suggested that the gut microbiome of long-lived animals such as reptiles should be investigated for their unique properties and contribution to the potent defense system of these species could be extrapolated for the benefit of human health. A range of techniques can be used to modulate the gut microbiota to have higher abundance of “beneficial” microbes that have been linked with health and longevity.

## 1. Introduction

The link between human health and the gut microbiome is profound and has been speculated upon for thousands of years. In 400 B.C., it was suggested by Hippocrates that “bad digestion is the root of all evil” and “death sits in the bowels” [1]. It is now well-known that humans are inhabited by microorganisms, including bacteria, viruses and archaea, that live in harmony with them, known as microflora, microbiota or normal flora [2,3]. It is estimated that the human microbiome is comprised of a hundred trillion bacteria cells, which amounts to ten times more than there are human cells in the human body [2,4]. With a surface area of 200 m^2^ to 300 m^2^ [5], the gut alone accounts for over 70% of the human microbiota [3]. During the average human lifespan, along with approximately 60 tonnes of food, a plethora of microorganisms from the environment pass through the human gastrointestinal (GI) tract, and this results in coevolution forming intricate and mutually beneficial relationships, leading to the formation of the “gut microbiota” [6]. The gut microbial composition develops concomitantly with the host from birth and is influenced by several factors, including genetic predisposition and nutrition [7]. Even within the gut microbiota, striking differences are observed at different locations such as the duodenum, ileum and colon [8]. In addition, alterations in the gut microbiota may be influenced by environmental factors, toxins, pathogens, diet and drugs [9]. While the gut microbiota is present in the digestive tract, its effect is not limited to that location and is even known to modulate brain development in mammals and later behaviour in adults [10]. Many aspects of human health are also influenced by the gut microbiota, as they may provide energy and nutrients to the host by aiding the digestion of nondigestible dietary components and can also contribute to inflammation, infection, gastrointestinal diseases, diabetes mellitus and obesity [11]. Interestingly, the composition of the human gut microbiota shifts with age, leading to influenced changes in the host’s health [12]. In this regard, the disturbance of the microbiome has been suggested as a new hallmark of ageing in the recent meeting held in Copenhagen on March 2022, in addition to the original nine hallmarks of ageing proposed by López-Otín and colleagues in 2013 [13]. Herein, we discuss how the gut microbiome develops, the possible ways of manipulating the gut microbiome and its role in disease and ageing. Furthermore, gut microbiota interventional strategies are discussed, as well as the notion of learning from long-lived animals such as reptiles.

## 2. Gut Microbiota Development and Composition

Formerly, it was thought that initial contact with microbial species takes place during birth, as was proposed by Tissier in 1900. The placenta was thought to prevent microorganisms from entering the bloodstream of the fetus, thus maintaining a sterile environment [14,15]. Nonetheless, recently it has emerged that diverse microbial communities may exist in human semen and in the womb [14,16]. Furthermore, a variety of other reports state that microbial species may inhabit diverse niches, comprising the placenta, umbilical cord blood and amniotic fluid, suggesting that select microbes may colonize in utero [14,17,18,19]. A recent study was conducted in mice whereby pregnant mice were colonized with genetically labelled *Enterococcus faecium*, that was isolated from human breast milk; the labelled strain was successfully cultured from the amniotic fluid of mice 2 days before full term was achieved [17]. However, even though the findings suggest transfer of some microbial species prior to birth, the results remain controversial because of the probability of contamination while assessing the specimens, and so prospective studies are necessitated [20]. Nonetheless, if this is the case, such microbial studies could be extrapolated and studied in order to determine their unique properties.

The human gut microbiota typically undergoes an age-affiliated microbiota shift from 3 days after birth to 2, 3, 4 or 5 years later when the gut microbiota reaches an adult-like configuration [21,22,23,24]. Effects of the microbiota on the host can be both beneficial and harmful, and while it is estimated that 40% of the microbiota is yet to be identified or cultured, links between the gut microbiota composition and human health have been established, even in infants [25]. For example, in infants, reduced numbers of bifidobacterial species have been linked to atopic sensitization and future development of obesity, while increased numbers of *Clostridium difficile* have been linked to colicky infants [26,27,28]. 

Initially, the gut microbiota is influenced by prenatal factors, including maternal diet, antibiotic use during pregnancy, maternal stress, smoking status and obesity [29,30]. The microbiota is then affected by the type of birth, mainly vaginally and through Caesarean section, whereby vaginally delivered children, as compared to those delivered through Caesarean section, displayed lower rates of asthma, atopic symptoms and diabetes, while interestingly, specific colonization of the microbiota in children delivered through Caesarean section was delayed by up to one 1 month and the diversity and number of colonies in their microbiota were lower [31,32,33,34]. Newborns are typically exposed to vaginal microbes that are typically dominated by Lactobacilli and Prevotella spp. [35]. The microbiome of babies born via Caeserean section is normally dominated by *Staphylococcus*, *Propionibacterium* spp. and *Corynebacterium* [35,36]. Whether modulation of these microbiomes at an earlier stage can affect ageing and the precise manner in which this may occur needs to be further investigated. For example, inoculation with maternal vaginal microbiota immediately following C-section delivery is denoted as “vaginal seeding” [37]. Nonetheless, there are limitations pertaining to this method as there is a lack of comprehension of the precise mechanisms in early microbiota initialization and maturation. A major limitation is the ability to study neonates in sufficient number. Moreover, the link between impaired maternal microbiota transfer at birth and acute or chronic illnesses in infants born by C-section needs to be determined, and future work is needed.

The admixture of compounds and antigens in breast milk, together with its range of bifidobacteria and lactobacilli strains, are the next factors that may affect the development of the microbiota [25]. A recent study revealed that having older siblings positively impacted bacterial diversity and richness in 18-month infants, inducing effects that are more pronounced than early-life infections [38]. Notably, the administration of antibiotics in infants also affects the development of their microbiome transiently or persistently [39]. Autochthonous bacteria—indigenous bacteria of the gut flora—inhibit the growth of allochthonous species—bacteria from water, food or other organs—through the production of specific substances, which is partly responsible for keeping the gut flora constant [40]. 

## 3. Gut Microbiota Modulation

**Figure 1:**
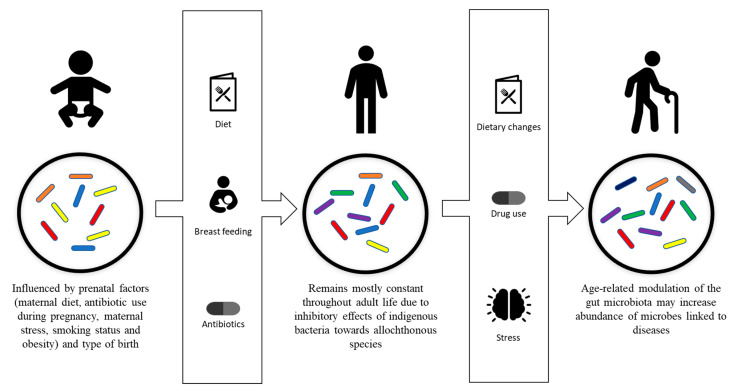
Development and modulation of the gut microbiota. Gut microbiota is initially influenced by prenatal factors, including maternal diet, antibiotic use during pregnancy, maternal stress, smoking status, obesity and type of birth. Breastfeeding, diet and antibiotic usage are the factors that affect the development of the gut microbiota into its stable adult composition. The adult composition of the gut microbiota may be modulated by drug usage, dietary changes, psychological stress and physiological stress.

While it is known that following initial colonization, the gut flora of humans remains mostly constant throughout their adult life [41], evidence of intestinal microbiota changes in adult humans have been reported and linked to specific phenomenon, including drug use, radiation, psychological stress, dietary changes, altered gastrointestinal tract peristalsis and physical stress (Figure 1) [1,42].

The most commonly used administration route for drugs is the oral route, owing to the fact that it allows for the uptake of drugs without medical intervention, which exposes the gut microflora to drugs [43]. For example, conventional anti-inflammatory drugs such as naproxen, ibuprofen and aspirin, when taken daily over months, affect intestinal microbial composition [44]. In addition, proton-pump inhibitors that are often taken in combination with anti-inflammatory drugs to reduce the formation of stomach ulcers have been reported to alter the gut microbiome [45]. The impact of drugs on intestinal microbes is not limited to anti-inflammatory drugs and has also been linked to other type of medications, such as the use of metformin in the treatment of type 2 diabetes, which has been shown to change the gut microbiota composition [46].

However, the most significant and common source of changes in normal gut microbiota has been linked to antibiotic use [47,48]. Pharmacokinetics, spectrum of activity, length of administration and dosage are some of the factors of antibiotic usage that influence the impact of the antimicrobial agent on the gut flora [1]. Antimicrobial agents may severely impact the gut microbiota, leading to an overgrowth of microorganisms, such as fungi, that are already present [47,49]; a decrease in the production of short-chain fatty acids, responsible for electrolyte and water absorption, leading to electrolyte imbalances [50,51]; and/or a decrease in colonization resistance by autochthonous bacteria, resulting in increased susceptibility to intestinal pathogens [52,53]. While most orally administered antimicrobial agents will trigger changes in the gut microbiota, the effects might be transient or long-lasting [43]. For example, some antibiotics, such as amoxicillin, do not have long-term impacts on the gut microbiome [54], while others, such as ciprofloxacin, leave a long-lasting signature [55]. Furthermore, in elderly people, it has been observed that repeated exposure to antibiotics may lead to destabilization of the gut flora resulting in antibiotic-resistant pathogenic bacteria outgrowth [56].

Moreover, it has been demonstrated that the integrity of indigenous microflora can be altered for several days due to psychological stress [57]. Changes in the gut microbiota of Soviet cosmonauts were also identified and linked primarily to stress and increased risk of colonization by pathogenic microorganisms [58]. It is now recognized that microgravity environments can affect gut microbial composition [59]. In addition, 20% to 30% changes in levels of some bacterial species were revealed in faeces of humans when experiencing anger or fear, and concentrations of the bacteria returned to normal after the resolving the situations [60]. Furthermore, a recent study that examined changes in the gut microbiome of a world-class ultramarathon runner before and after competing revealed shifts in human gut microbiome composition after acute exercise [61]. In another study, it was demonstrated that gut microbial modulation with probiotics during a stressful ship voyage to Antarctica was able to regulate gut microbiota homeostasis and reduce sea sickness prevalence as well as other physiological complications [62].

Nutrition can also affect the gut microbiota, as variations in food intake may cause both transient changes and cause shifts in specific bacteria leading to typical signatures [63,64]. It has been shown that the level, both low and high, of uptake of animal proteins and amino acids modulates the levels of certain bacteria and increases the activity of certain bacterial enzymes [65,66,67]. The amount of carbohydrates or sugars in regimes also affects the gut microbiota [1,43]. Food rich in sulphur also modulates the microbiota by promoting the growth of sulphate-reducing bacteria [68]. Fibres in food also have a predominant role in variation of the gut microbiota, since they are indigestible and cause microbial fermentation [43]. Moreover, it has been shown that a Western diet, comprising of higher fat/higher sugar and higher processed food intake, can lead to gut microbial changes [69,70].

## 4. Gut Microbiota and Disease

Gut microbiota dysbiosis has been clearly associated with the development of colorectal cancer [71], which has been linked to increased abundance of Firmicutes and Fusobacteria from 0.1% of the gut microbiota in healthy individuals to approximately 10% in colorectal cancer patients [72,73,74]. Changes in gut microbiota may lead to a population with higher carcinogenic potential, such as *Fusobacterium nucleatum*, bacteria that express Fusobacterium adhesin A, a virulence factor that activates growth and proliferation of cells, and is abundant in tumour tissues [75,76,77]. Another bacterium, *Bacteroides fragilis*, also stimulates cell proliferation and damages DNA through reactive oxygen species [78,79].

Increased levels of *Enterobacteriaceae* have been reported in elderly human subjects suffering from *Clostridium difficile*-associated diarrhoea [80,81], while it has been reported that by preferentially metabolizing carbohydrates that are essential for the growth of *Clostridium difficile*, the gut microflora suppresses the growth of *Clostridium difficile* [82,83]. Modulation of the gut microbiome may also lead to *Clostridium difficile*-associated colitis [84]. In addition, a decrease in the abundance of Firmicutes and increase in the abundance of Proteobacteria has been linked to necrotizing enterocolitis, a gut inflammation with a mortality of 30% [43]. Moreover, ulcerative colitis has been linked to a loss of bacterial diversity in the gut microbiota and an increase in the abundance of *Bacteroides* and *Candida* [85].

Recent studies have linked the bidirectional gut microflora–brain interactions to mood disorders, and this biochemical signalling has been termed the gut–brain axis [86,87]. Patients suffering from autism spectrum disorders exhibited increased abundances of *Lactobacillus*, *Corynebacterium* and *Collinsella* in the gut microbiota compared to unaffected siblings [88]. In individuals with bipolar disorder, higher levels of faecal *Clostridium*, *Oscillibacter*, *Bacteroides*, *Streptococcus* and *Bifidobacterium* were identified [87].

Intestinal microbiota of rheumatoid arthritis patients was found to be different from controls, and *Yersinia*, *Salmonella*, and *Shigella* have been linked to arthritis as triggers [89]. Gut microbiota changes are often reflected by a varying number of microorganisms, namely *Proteus mirabilis*, in the faeces of patients [90,91] and an increase in *Prevotella copri* [92]. In addition, specific changes in the gut microbiota of obese patients have been reported, such as a decrease in the abundance of *Bacteroides* spp [93,94] and an increase in the abundance of *Akkermansia muciniphila*, a mucin-degrading gut bacterium that, in obese patients, has been linked to enhanced metabolic health [95]. Moreover, diminished gut microbial diversity arising from vancomycin treatment and high levels of coliforms and *Staphylococcus aureus* has been linked to allergy [96,97].

## 5. Gut Microbiota and Ageing

Considering that the gut microbiota is modulated by various phenomenon, as described above, it is obvious that the gut microbiota changes throughout the lifespan of the human life, and hence at older ages the changes will become more visible. A study with 528 individuals aged 0 to 70 years demonstrated that there is a shift in the gut microbiota with age [22]. As documented in the previous section, it is apparent that alterations in the gut microbiota are linked with several health conditions.

Data from 371 samples, from subjects of an age range of 0 to 104 years, were analysed and classified into 187 bacterial groups, and 4 predominant phyla, namely Actinobacteria, Bacteroidetes, Firmicutes and Proteobacteria, were revealed [12]. The relative abundance of Actinobacteria decreased with age while the abundance of Firmicutes increased from children to adults and then decreased in elderly individuals, and the abundance of Proteobacteria and Bacteroidetes increased in individuals of 70 years and older [12]. *Bacteroides*, *Eubacterium* and Clostridiaceae co-abundance groups were associated with elderly groups; and co-abundance groups *Megamonas* and *Peptoniphilus* were found to be enriched in elderly [12]. Betaproteobacteria, Bacteroidetes and Deltaproteobacteria were found in higher abundance in the elderly, as compared to infants, adolescents and adults [12].

Moreover, gut microbiota of 84 subjects, aged 25 to 104 years, were analysed using the Human Intestinal Tract Chip and quantitative PCR [98]. The study revealed that *Enterobacter aerogenes*, *Eggerthella Iento*, *Bacillus*, *Clostridium leptum* and *Clostridium orbiscindens* abundance gradually increased from young individuals to centenarians while an increase in the abundance of *Eubacterium limosum*, *Klebsiella pneumoniae*, *Vibrio*, *Sporobacter termiditis* and *Anaerotruncus colihominis* was identified in centenarians as compared to elderly [98]. Interestingly, the abundance of *Clostridium colinim*, *Clostridium sphenoides*, *Eubacterium rectale*, *Lachnobacilus bovis*, *Ruminococcus actaris* and *Ruminococcus obeum* were higher in elderly as compared to young individuals but was shown to decrease in centenarians as compared to elderly [98]. Conversely, a gradual decrease in the abundance of *Faecalibacterium prausnitzii*, *Papillibacter cinnamovorans*, *Eubacterium hallii*, *Eubacterium ventriosum* and *Roseburia intestinalis* was observed from in centenarians and elderly as compared to young individuals [98].

Similarly, gut microbiota of 24 semisupercentenarians, aged 105 to 109 years, and 15 young adults, aged 22 to 48 years, were analysed [99]. The study also included precollected gut microbiota of 15 centenarians, aged 99 to 104 years, and 15 elderlies, aged 65 to 75 years [99]. An increase in the abundance of *Anaerotruncus*, *Odoribacter*, *Oscillospira*, *Bilophila* and *Eggerthella* was observed in gut microbiota as age increased from young adults to semisupercentenarians, through elderly and centenarians [99]. On the other hand, a decrease in the abundance of *Coprococcus* and *Roseburia* was observed as age increased from young adults to semisupercentenarians [99]. Interestingly, the abundance of *Christensenellaceae*, *Butyricimonas* and *Barnesiellaceae* increased as age increased from adults to centenarians, but then decreased from centenarians to semisupercentenarians [99]. The abundance of *Lachnospiraceae* and *Akkermansia* increased from young to elderly, then decreased from elderly to centenarian and then showed an increase from centenarians to semisupercentenarians [99]. The abundance of *Faecalibacterium* was shown to increase from adults to elderly and then decrease [99].

In another study, gut microbiota of 24 subjects, aged 100 to 108 years (centenarian), 85 to 99 years (elderly) and 80 to 92 years (younger elderly), were analysed [100]. It was revealed that as age increases, the relative abundance of *Verrucomicrobiaceae*, *Veillonellaceae*, *Porphyromonadaceae*, *Barnesiellaceae*, *Odoribacteraceae* and *Alcaligenaceae* decreases [100]. As age increased from younger elderly to elderly, the relative abundance of *Methanobacteriaceae*, *Lactobacillaceae* and *Comamonadaceae* increased; but when age increased from elderly to centenarian, their relative abundance decreased [100]. In addition, the relative abundance of *Catabacteriaceae* decreased from younger elderly to elderly and increased from elderly to centenarian [100]. Relative abundance of *Enterobacteriaceae* increased from younger elderly to elderly while that of *Rikenellaceae* decreased from elderly to centenarian [100].

Interestingly, a study analysed the gut microbiota composition of 168 participants (67 healthy centenarians, 54 elderly and 47 young adults) from China and compared the results with a dataset of Italian participants [101]. It was revealed that the relative abundance of *Clostridium*, *Ruminococcaceae*, *Akkermansia* and *Christensenellaceae* are enhanced in the older groups in both the Chinese and Italian populations [101]. This indicates that the age-related modulation of the gut microbiota might be a process that takes place regardless of geological location.

A recent study, comprising data from more than 9000 individuals, demonstrated that the uniqueness of the microbiome that arises with age has a strong link to amino acid derivatives produced by microbes that circulate in the bloodstream [102]. It was observed that the gut microbiome of healthy individuals that are over 80 years still evolves towards a unique compositional state, while this change is not apparent in less healthy individuals [102]. Interestingly, decreased survival was observed in individuals with a high abundance of *Bacteroides*, a fundamental genus observed in most humans, and those displaying a low gut microbiota uniqueness [102].

## 6. Gut Microbiota and Age-Related Disease

Ageing alters the gut microbiota, as shown above, and this age-related modulation of the gut microbiota may be a major contributor to poorer outcomes after acute injury in the elderly and incidence of numerous age-related diseases [103]. While ageing is a complex process that involves numerous biological pathways, age-related chronic inflammation, notably with regards to its links with degenerative disorders and frailty, is believed to be pathogenic, and hence, inflammation-mediated aging (inflammaging) has been proposed as a vital mechanism of aging [104]. It has been suggested that age-related dysbiosis of the gut microbiota might in turn mediate age-related chronic inflammation [105]. Moreover, it has been shown that the gut microbiota is essential in both the development of immune maturity in the gut and also systemic immunity, and additionally, it modulates the architectural organization of secondary lymphoid organs into distinct T-cell and B-cell zones [106]. This is further supported by the fact that a decrease in the abundance of lactobacilli and bifidobacterial, bacterial species that may cause upregulation of interleukin-10 and downregulation of tumour necrosis factor-α and interferon-γ, is observed in the elderly [105].

Moreover, *Faecalibacterium prausnitzii*, commonly found in the human gut, is known to exert an anti-inflammatory effect on the gut mucosa, and a decrease in its abundance has been linked to the trigger of Crohn’s disease [107]. Presence of *F. prausnitzii* has also been linked to a reduction in the severity of colitis in animal models [107]. Upregulation of interleukin-10 and downregulation of interleukin-12 and interferon-γ has been demonstrated in peripheral blood mononuclear cells exposed to *F. prausnitzii* [107]. Interestingly, the abundance of *F. prausnitzii* is diminished in the gut microbiota of elderly, and the loss of abundance of *F. prausnitzii* in centenarian gut microbiota has been linked with increased levels of proinflammatory cytokines interleukin-6 and interleukin-8 in the blood [98]. It has been further demonstrated that a lower abundance of *F. prausnitzii* is observed in stool samples from hospitalized elderly as compared to nonhospitalized elderly [108], further indicating that decrease in its abundance contributes to poor health.

The regulation of skeletal muscle mass is influenced by muscle protein synthesis and breakdown, and in older individuals, the sensitivity of muscle protein synthesis to anabolic stimuli, when compared to younger individuals, is weakened [109]. This diminished anabolic responsiveness is termed anabolic resistance [109]. Gut microbes play an important role in the digestion, processing and absorption of protein in the gastrointestinal tract [110]. In addition, microorganisms such as Bifidobacteria and Clostridia have been shown to produce amino acids from nonspecific nitrogen sources [111]. Moreover, 19% to 22% of leucine, an amino acid that may be a requirement to initiate anabolic intracellular signalling pathways, is synthesized by intestinal microbes [112,113]. Interestingly, bacteria such as *Prevotella*, which are involved in the biosynthesis of lysine (an amino acid that is essential for muscle protein turnover that has also been linked to longevity when present in high levels) have been shown to decrease in abundance with human ageing [109]. Similarly, a reduction in abundance of *Lactobacillus* with advancing age has been observed, while *Lactobacillus plantarum* has been shown to be important in the expression of insulin-like growth factor 1, which is known to affect muscle development and growth throughout life [109]. Hence, age-related gut dysbiosis may play a predominant role in age-related anabolic resistance.

A study using animal models showed that transplantation of gut microbiota from older mice into younger mice increased mortality following induced ischemic stroke as compared to young mice with unaltered gut microbiota [103]. This indicates that the ageing gut microbiome might be partly responsible for poor outcomes following stroke in the elderly. Further studies into the comprehension of the mechanism utilized by gut microbiota are needed.

## 7. Gut Microbiota Interventional Strategies

**Figure 2:**
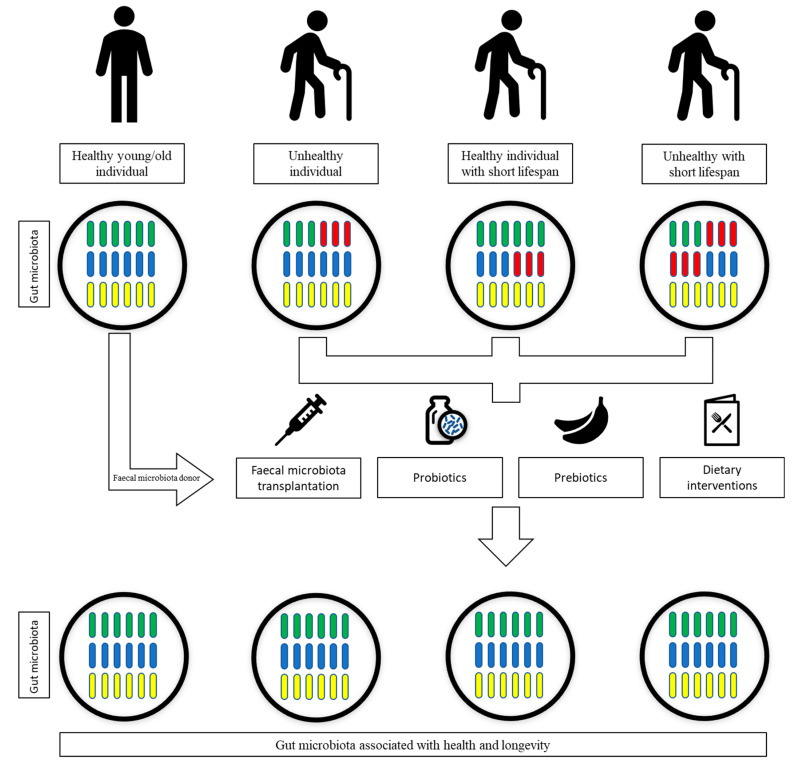
Voluntary modulation of the gut microbiota to improve health and longevity. Gut microbiota composition and abundance of specific microbes have been linked to health and longevity. Faecal microbiota transplant, prebiotics usage, the use of probiotics and dietary interventions can be exploited to modulate the gut microbiota to have higher abundances of microbes linked with health and longevity.

It is clear that the age-related dysbiosis of the gut microbiota may lead to unhealthy ageing, contribute to the development of comorbidities and may even dictate the lifespan of individuals, as shown in the previous sections. Hence, by inducing changes in the gut microbiota, it might be possible to improve the health of the elderly and even prolong their lifespan (Figure 2). A recent a randomized controlled clinical trial was conducted, which comprised analysis of diet, sleep and exercise, as well as supplementation with probiotics and phytonutrients [114]. The trial revealed that these lifestyle changes resulted in an approximately 3-year decrease in epigenetic age using the using the online Horvath DNAmAge clock in comparison to control subjects [114]. Future trials should focus on the change in the gut microbiome as well, to identify particular changes in gut microbial diversity and species of interest. Although a plethora of studies have examined the onset of unhealthy ageing and the microbiome of rodents [115], a major limitation is whether such studies may be extrapolated to humans. Furthermore, the cohort size or number of participants is an important factor of consideration in current studies that are ongoing in humans [116]. The mean number of participants involved in studies with a focus on ageing and older participants is approximately 36, and in some cases such studies involve only few patients [116]. A further shortcoming to consider is that some of these studies may utilize low-resolution microbiome profiling or rely on the changes in the host physiology rather than the microbiome profiles [116]. There is also the risk of transmission of microbiota, which may detrimental to older patients [117]. 

### 7.1. Dietary Interventions 

Diet can be used to modulate the composition of gut microbiota. An abundance of short-chain fatty acids, which are produced by gut microbes and have been shown to exhibit protective roles against a panoply of diseases, has been shown to correlate with diets [118]. It was shown that as compared to an animal-based diet, vegan and vegetarian diets which comprise—through fruit and vegetables—greater intake levels of fibre, resulted in higher levels of short-chain fatty acids [118]. The microbiota associated with vegetable-based diets correlate with short-chain fatty acid levels, and *Prevotella* and *Lachnospira* are thought to be bacteria that lead to the increased production of short-chain fatty acids [118].

The structure of the gut microbiota and microbial gene expression was shown to be altered by the short-term consumption of diets made up entirely of plants or entirely of animal products [70]. It was determined that by switching to a carnivorous diet from a herbivorous one, the gut microbiota composition, activity and metabolic pathways may be modulated within a single day [70]. Abundances of *Alistipes*, *Bilophila* and *Bacteroides* were increased in individuals under the animal-based diet, while abundances of *Roseburia*, *Eubacterium rectale* and *Ruminococcus bromii*, involved in the metabolism of dietary plant polysaccharides, were decreased [70].

It has also been hypothesized that coffee consumption may modulate the gut microbiota composition, resulting in protection against Parkinson’s disease [119]. The abundance and activity of *Bifidobacterium*, (bacteria that possess anti-inflammatory properties) are increased by coffee consumption, which mitigates the gastrointestinal inflammation (an event that has been demonstrated in the early stage of Parkinson’s disease) [119]. In turn, this reduces the misfolding of α-synuclein in enteric nerves, thereby reducing the risks of Parkinson’s disease by diminishing neurodegeneration that would be induced by propagation of the protein aggregates [119]. Additionally, intake of coffee might promote the abundance and activity of microbes that counteract gastrointestinal infection, such as *Helicobacter pylori* infection, which are overrepresented in Parkinson’s disease patients [119].

Interestingly, calorie restriction is known as the only experimental procedure that can, in various animal models, effectively lengthen lifespan [120]. In a study that studied the shift in gut microbiota induced by a high-fat diet versus a low-fat diet in mice, it was demonstrated that mice with a 30% restriction of low-fat diet, as compared to the ad libitum group, had a unique gut microbiota, indicating that modulation of the gut microbiota can be achieved only by restricting the intake of diet [120]. Moreover, calorie restriction was shown to enrich phenotypes that have been positively associated with lifespan, and decrease those that correlate negatively with lifespan [120]. The gut microbiota modulations induced by calorie restriction were also associated with a reduction in lipopolysaccharide-binding proteins, indicating that gut microbiota architecture established through calorie restriction can exert a health benefit [120].

In summary, diet can be used to modulate the composition of gut microbiota, which may be tweaked to increase the abundance of microbes associated with a longer lifespan while inducing a decrease in the abundance of microbes that negatively correlate with lifespan.

### 7.2. Prebiotics and Probiotics

Prebiotics—nondigestible food ingredients that are metabolized by selective intestinal microorganisms—can be used to modulate the gut microbiota to increase the abundance and activities of beneficial bacteria [121]. Oligosaccharides, nondigestible carbohydrates and short polysaccharides, including inulin, galactooligosaccharides, galactofructose, oligofructose and xylooligosaccharides, are common components of prebiotics [122]. Prebiotics resist digestion in the small intestine to reach the colon, where they are acted upon by gut microflora, leading to specific changes in composition and activity in the gut microbiota [123].

Probiotic supplementation has been applied in the modulation of the gut microbiota to convey health benefits. Probiotics are defined as live microorganisms that confer to improve the health of the host when adequate amounts are administered in a safe and efficacious manner [124]. Several studies have supported the use of probiotic supplementation for its therapeutic effects against a broad range of diseases, especially for metabolic and gastrointestinal disorders [125]. In addition, the ability of probiotic supplementation to modulate the gut microbiota was reported in terms of faecal bacterial community structure being significantly different as compared to placebo [126]. One of the main proposed mechanisms of action of probiotics is the modulation of gut microbiota abundance in favour of desirable species with known health benefits, but probiotics may also act by competing with harmful species for adhesion sites (for example, *Escherichia coli* Nissle can displace other microbes and prevent their adhesion) by producing antimicrobial compounds (for example, reuterin produced by *Lactobacillus reuteri* possesses antimicrobial activity) or by inducing immune responses [122].

A study assessing the possible effects of probiotics in acute infectious diarrhoea, encompassing 12,127 participants, revealed that the duration of hospitalization of individuals using probiotics was shorter compared to the control group [127]. Antibiotic-associated diarrhoea, often caused by *Clostridium difficile*, may be reduced by using probiotics [128]. The efficacy of probiotics in the treatment of chronic inflammatory bowel disease has also been investigated, and reports indicate that probiotics are as effective as usual treatment in the prolongation of remission and reduction of symptoms [129]. The ability of probiotics in the relief of irritable bowel syndrome was also analysed, through information from 1650 patients, and it was revealed that probiotics may be used as an alternative treatment, considering how they showed significant effects as compared to the placebo group [130].

In summary, probiotics have shown their capability in the treatment of gut-related diseases, and are thought to work through modulation of the gut microbiota. Prebiotics are employed specifically for the modification of gut microbiota. Hence, probiotics and prebiotics might be utilized to promote the abundance of gut microbes that have been associated with health and longevity.

### 7.3. Faecal Microbiota Transplantation

Faecal microbiota transplantation is the administration, into a recipient’s intestinal tract, of the whole microbiota from healthy donor faeces to modify or normalize intestinal microbiota composition [131]. The ability of faecal microbiota transplantation to treat several diseases, including irritable bowel syndrome, metabolic diseases, autoimmune diseases, constipation, neuropsychiatric conditions, colon cancer, chronic fatigue syndrome and allergic disorders, has been reported [122]. The ability of faecal microbiota transplantation to treat *Clostridioides difficile* infection in the elderly (85 years old and above) was recently investigated, and the report indicated that severe infections in all cases were improved following one faecal microbiota transplantation, indicating that “frail older people” might benefit from faecal microbiota transplantation [132].

Recently, capsules and enteric-coated capsules containing freeze-dried bacteria or faeces were used in faecal microbiota transplantation, and were shown to be highly efficient [122]. However, several pathways have been utilized in the transplantation of faecal microbiota. Transplantations of faecal suspensions from donors, screened for pathogens, were administered via retention enemas in patients suffering from ulcerative colitis and were shown to be effective in the reversal of symptoms and resulted in patients free from ulcerative colitis up to 13 years post-treatment [133]. Furthermore, in a study with patients of a mean age of 69.4 years old, it was demonstrated that the administration of faecal suspension from healthy donors through enema can be used to successfully treat *Clostridium difficile* infection [134]. Similarly, another study studied the effects of self-administration of faecal suspension, from family donors tested for pathogens and comorbidities, using low-volume enema in the treatment of *Clostridium difficile* infection [135]. With up to 14 months’ follow-up, 100% clinical success was observed, and no repeat procedure was required for any patients, indicating that faecal transplantation by low-volume enema at home is an effective and safe option [135].

Bowel lavage by macrogol followed by faecal transplantation, in the form of fresh faeces from healthy donors through colonoscopy into the caecum, was used in the treatment of *Clostridium difficile* infection in patients (mean age of 70) and it was revealed as an effective cure [136]. Similarly, colonic lavage through polyethylene glycol solution, followed by faecal transplantation of homogenized donor faeces into the caecum through colonoscopy using a biopsy channel in patients (mean age of 73) suffering from *Clostridium difficile* infection was found to be an effective treatment [137]. More recently, the efficacy of faecal microbiota transplantation in the treatment of ulcerative colitis was investigated by administration, through colonoscopy into the 40 cm proximal of terminal ileum, of filtered faecal microbiota suspension extracted from fresh faecal suspension, following bowel lavage using sennoside, calcium and water [138].

Faecal transplantation of filtered faecal samples into the stomach of patients (mean age of 73) through a nasogastric tube, placed in the stomach using radiography for the tip placement position, was found to be effective in the treatment of *Clostridium difficile* infection [139]. Another study also demonstrated the ability of faecal microbiota transplantation by nasogastric tube into the upper gastrointestinal tract of patients in the treatment of *Clostridium difficile* infections [76]. Faecal microbial transplant in the treatment of Crohn’s disease was investigated by transferring homogenized faecal samples into patients through a nasogastric tube, and it was revealed as an effective treatment [140]. In another study, faecal microbiota was purified through centrifugation and filtration and was then transferred to patients suffering from ulcerative colitis, and was shown to be a therapeutic strategy, which is likely due to the restructuration of gut microbial composition [141]. A nasoduodenal tube has also been used for the transplantation of faecal material into the duodenum following bowel lavage in the treatment of *Clostridium difficile* infection, and was shown to be more effective than vancomycin [142]. 

In summary, faecal microbiota transplantation is a method that has extensively been utilized to “normalize” gut microbiota to tackle infection, such as *Clostridium difficile* infection. Hence, faecal microbiota transplantation may be used to modulate gut microbiota to promote the abundance of gut microbes that have been associated with health and longevity; however, more research is needed. Nonetheless, the risk of transmission of microbiota that may detrimental to older patients needs to be examined and is a limitation to be mindful of [117].

## 8. Long-Lived Animals and Gut Microbiome, Can We Learn from Them? 

Ageing is possibly one of the key biological challenges affecting human health; thus, comprehending the precise biological mechanisms is warranted [143]. The use of long-lived animals may offer distinctive prospects to identify the mechanisms, processes and genes that offer protection against ageing [144]. In this regard, reptiles are certainly a remarkable species, an example being crocodiles, which have been designated as ‘living fossils’ [145]. Reptiles are thought to have originated in the last 310–320 million years, and the crocodilians endured mass-extinction events, such as the Cretaceous–Tertiary extinction event [146]. On the other hand, *Homo sapiens* are simply one species amongst millions in this planet, and compared to other species, are a relatively new addition. Thus, it makes sense to investigate the defense system of long-lived animals such as reptiles who exhibit the abilities to evolve, adapt and survive successfully over millions of years, dwelling in often harsh environments containing heavy metals and coming across radiation [147]. Given that most of the immune system is thought to be present in the gut, accordingly it has been recently postulated that the gut microbiome of reptiles most likely contributes to their robust immune system, their ability to thwart infectious diseases and cancer, and in some cases display limited or negligible senescence [148].

It is now apparent that reptiles such as crocodiles possess a unique gut microbiome, and this likely influences their ability to produce antimicrobial peptides, as well as their apparent resistance to infection and cancer and their longevity [149,150]. Recently, it was shown that selected gut bacterial metabolites from the saltwater crocodile exhibited potent activities against various cancer cell lineages, and numerous molecules were elucidated, which could be investigated for their effects on ageing and longevity. Nonetheless, at present, very few studies on the reptile gut microbiome, and even less research on reptile gut microbial metabolites, are underway. Future studies to understand effects of these gut microbial metabolites from reptiles into models of disease or ageing/germ-free in vivo models should be warranted, and may offer an alternative approach for the development of novel interventions to modulate the ageing gut microbiome.

## 9. Concluding Remarks

The lifespan of individuals and healthy ageing has been linked to the gut microbiota composition, and hence, the health of the elderly and their lifespan might be improved through modulation of the gut microbiota. Interestingly, it has been demonstrated that dietary interventions, prebiotics, probiotics and faecal microbiota transplantation may be utilized to modulate the gut microbiota. In terms of dietary interventions and prebiotics, controlling these to promote microbes that are beneficial to health or have been linked to longevity is desired, and needs to be carefully investigated. Probiotics and faecal microbiota transplantation might be exploited to shift the composition of the gut microbiota of the elderly to resemble those of younger individuals or healthier elderly. Additionally, strategies for the safe and efficient delivery of faecal microbiota, probiotics or specific microbes into the gut microbiota need to be developed, especially in older patients. Furthermore, studies with well-defined probiotic strains should be utilized, and should also consider combinations of various probiotics and metabolites, as well as dosage, timing of administration and the formulation of the product. Finally, the use of long-lived species such as reptiles should be investigated for their gut microbial metabolites, to understand the mechanisms of longevity and ageing in these robust species, and this may offer the discovery of exciting and innovative molecules, which may be used for the benefit of human health and human performance.

## Data Availability

Not applicable.
